# Anti-Inflammatory Effects and Decreased Formation of Neutrophil Extracellular Traps by Enoxaparin in COVID-19 Patients

**DOI:** 10.3390/ijms23094805

**Published:** 2022-04-27

**Authors:** Supichcha Saithong, Wilasinee Saisorn, Punyot Tovichayathamrong, Grace Filbertine, Pattama Torvorapanit, Helen L. Wright, Steven W. Edwards, Asada Leelahavanichkul, Nattiya Hirankarn, Direkrit Chiewchengchol

**Affiliations:** 1 Department of Microbiology, Faculty of Medicine, Chulalongkorn University, Bangkok 10330, Thailand; supichcha_mumt@hotmail.com (S.S.); wsaisorn@gmail.com (W.S.); punyot.tovi@gmail.com (P.T.); dr.gracefilbertine@gmail.com (G.F.); aleelahavanit@gmail.com (A.L.); 2 Translational Research in Inflammation and Immunology Research Unit (TRIRU), Faculty of Medicine, Chulalongkorn University, Bangkok 10330, Thailand; 3 Center of Excellence in Immunology and Immune-Mediated Diseases, Faculty of Medicine, Chulalongkorn University, Bangkok 10330, Thailand; 4 Institute of Life Course and Medical Sciences, University of Liverpool, Liverpool L69 7ZX, UK; hlwright@liverpool.ac.uk; 5 Thai Red Cross Emerging Infectious Diseases Clinical Center, King Chulalongkorn Memorial Hospital, Bangkok 10330, Thailand; ptorvorapanit@gmail.com; 6 Institute of Infection, Veterinary and Ecological Sciences, University of Liverpool, Liverpool L69 7ZX, UK; s.w.edwards@liverpool.ac.uk

**Keywords:** neutrophil extracellular traps, COVID-19, enoxaparin, heparin, anti-inflammation

## Abstract

Neutrophil Extracellular Traps (NETs) are a contributing factor of vascular thrombosis and alveolar damage in COVID-19 patients. As enoxaparin is currently used to inhibit vascular thrombosis, this study aimed to investigate whether enoxaparin also reduced inflammation and NETs in COVID-19 patients. Patients with COVID-19 infection were classified into three groups: mild, moderate, and severe (*n* = 10 for all groups). Plasma was collected from patients and healthy donors (*n* = 10). Neutrophils isolated from healthy controls were incubated with COVID-19 or healthy plasma, and with or without enoxaparin pretreatment in vitro. Neutrophils and plasma isolated from patients treated with enoxaparin were also investigated. The levels of inflammatory cytokines and NET products such as dsDNA, NE, MPO–DNA and Histone–DNA complexes in plasma and supernatants were measured using immunofluorescence staining and ELISA kits. The expression of inflammatory signaling genes by neutrophils (*RELA*, *SYK*, *ERK* and *PKC*) was measured using real-time qPCR. The levels of NET products were elevated in the plasma of COVID-19 patients, particularly in the severe group (*p* < 0.01). Moreover, plasma from the severe group enhanced NET formation (*p* < 0.01) from neutrophils in vitro. Enoxaparin pretreatment in vitro decreased plasma-induced NETs in a dose-dependent manner and down-regulated the expression of inflammatory genes (*p* < 0.05). Patients treated with prophylactic enoxaparin showed lower inflammatory cytokine levels and expression of inflammatory genes (*p* < 0.05). Increased NETs were associated with the severity of COVID-19 infection, particularly in patients with severe pneumonia, and could be used as biomarkers to assess disease severity. Enoxaparin pretreatment inhibited NETs and reduced the expression of inflammatory cytokines, and these effects mostly persisted in patients treated with prophylactic enoxaparin.

## 1. Introduction

The COVID-19 pandemic has become a major problem worldwide. This airborne contagious disease is caused by the SARS-CoV2 virus that infects the cells of both the upper and lower respiratory tracts. The symptoms of patients with COVID-19 infection are varied, ranging from asymptomatic or mild symptoms to severe pneumonia or acute respiratory distress syndrome (ARDS). The lungs are the most affected organ because type II alveolar cells highly express angiotensin-converting enzyme 2 (ACE2) receptors on their cell surface, which binds to the COVID-19 spike protein and allows the virus to enter the host cells [1,2]. Recent studies have shown that a large number of patients develop severe respiratory symptoms, leading to high morbidity and mortality rates after COVID-19 infection [3].

The pathophysiology of the lower respiratory tract of patients with severe COVID-19 infection has demonstrated numerous inflammatory cell infiltrates, particularly neutrophils, which are recruited to the site of infection and cause lung damage [4,5,6]. Previous studies showed significant dynamic hematological changes of COVID-19 patients, including elevated neutrophil to lymphocyte ratios (NLR), which are strongly correlated with disease severity and progression [7,8]. Moreover, inflammatory markers of neutrophils (e.g., cytokines, enzymes, and antimicrobial peptides), including the levels of neutrophil extracellular traps (NETs), were significantly increased in both the serum and lung tissue of severe cases of COVID-19 infection [9,10,11].

Abnormal coagulation is another contributing factor to the pathogenesis of lung injury in COVID-19 patients with severe pneumonia. It was found that increased D-dimer and fibrinogen levels and prolonged prothrombin time were observed in severe cases of COVID-19 infection, leading to thromboembolic vascular complications in multiple organs [12]. Therefore, one treatment option in cases with severe pneumonia is the administration of anticoagulant prophylaxis, such as low-molecular-weight heparins (e.g., enoxaparin). According to the American Society of Hematology (ASH) guidelines, it has been suggested that severe hospitalized COVID-19 patients without a history of bleeding disorders but suspected or confirmed with venous thromboembolism (VTE) should be considered for enoxaparin prophylaxis [13].

The actions of enoxaparin on blood coagulation and anti-inflammation include: (a) the prevention of thromboembolic complications [14], (b) inhibition of SARS-CoV2 entry into the host cells, and (c) prevention of inflammatory cytokines and inflammatory proteins binding endothelial or epithelial receptors [15]. Moreover, a recent study has shown that enoxaparin interferes with neutrophil autophagy, granule redistribution and chromatin decondensation [16]. Since NETs and the inflammatory markers of neutrophils play a role in the pathogenesis of both lung injury and intravascular thrombosis in COVID-19 patients, this study therefore investigated whether enoxaparin treatment: (a) prevented NETs induced by COVID-19 infection and (b) decreased inflammatory markers in COVID-19 patients.

## 2. Results

### 2.1. Demographic Data

In total, 30 patients diagnosed with COVID-19 infection were divided into three groups: mild symptoms (*n* = 10), moderate pneumonia (*n* = 10), and severe pneumonia or ARDS (*n* = 10). Table 1 shows the demographic data of these COVID-19 patients. The percentages of females were: 42.6% with mild disease; 33.3% with moderate disease; and 16.7% with severe disease. The mean age was 32.7 years with mild disease, 50 years with moderate disease and 48.6 years with severe disease. There were no significant differences in mean weight, mean height, and mean body mass indexes among groups. Underlying diseases were found in patients with moderate to severe disease, particularly diabetes, hypertension, dyslipidemia, and chronic kidney disease in severe cases.

The laboratory findings showed no differences in the counts of red blood cells, white blood cells or platelets among groups (Table 2). However, the serum levels of the liver enzymes AST (*p* = 0.004) and ALT (*p* = 0.04), creatinine (*p* = 0.04) and bicarbonate (HCO_3_^−^) (*p* = 0.03) were significantly higher in the severe group when compared to the other groups. Moreover, the blood coagulation function showed significantly increased levels of D-Dimer (*p* = 0.02) and prothrombin time (*p* = 0.01) in the severe group (Table 3 and Table 4).

### 2.2. Increased NET Products in Plasma of COVID-19 Patients

**Figure 1:**
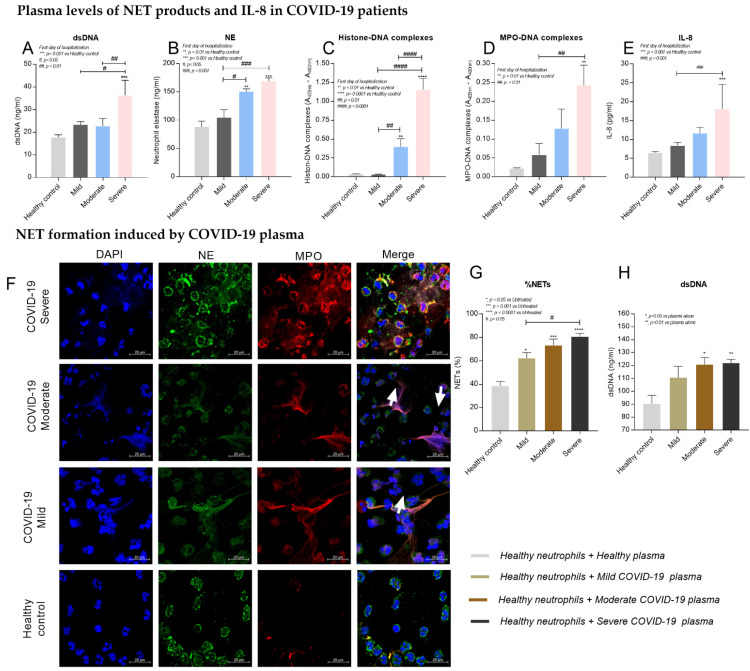
Plasma levels of NET products and IL-8 in COVID-19 patients and NET formation induced by COVID-19 plasma. The levels of extracellular double-strand DNA (dsDNA) (**A**), neutrophil elastase (NE) (**B**), Histone–DNA complexes (**C**), MPO–DNA complexes (**D**) and IL-8 (**E**) in COVID-19 patients with mild symptoms, moderate pneumonia, severe pneumonia, and healthy controls (*n* = 10 for all groups). Representative fluorescence images captured by confocal microscopy (original magnification 630Xand scale bars indicated object size as labelled 20 μm) of isolated neutrophils from healthy controls incubated with COVID-19 plasma or healthy control plasma (*n* = 10 for all groups), stained with DAPI (blue), neutrophil elastase (green) and myeloperoxidase (red), and merged images for NET identification (**F**). The percentages of NETting neutrophils (**G**) and the levels of dsDNA (**H**) from healthy neutrophils incubated with COVID-19 plasma or healthy control plasma (Supplemental Table S1). (* *p* < 0.05, ** *p* < 0.01, *** *p* < 0.001, **** *p* < 0.0001 vs. healthy control and # *p* < 0.05, ## *p* < 0.01, ### *p* < 0.001, #### *p* < 0.0001 vs. the indicated group).

The levels of NET products in plasma were compared among groups of COVID-19 patients and healthy controls. The results showed that the plasma levels of extracellular dsDNA (Figure 1A), NE (Figure 1B), Histone–DNA (Figure 1C), and MPO–DNA complexes (Figure 1D) were significantly increased in COVID-19 patients, particularly those with severe pneumonia (*p* < 0.01). There were no significant differences in the plasma levels of NET products between patients with mild symptoms and healthy controls. As IL-8 promotes NETs and is detected in COVID-19 patients [17,18], the levels of IL-8 in plasma were therefore investigated. The results showed that the plasma levels of IL-8 were significantly increased in severe cases when compared to patients with mild symptoms (*p* < 0.0001) and healthy controls (*p* = 0.0001) (Figure 1E).

### 2.3. Plasma of COVID-19 Patients Induced NETs

We then investigated whether the plasma of COVID-19 patients could induce NETs in vitro, as it has been reported that factors in the plasma of these patients contained SARS-CoV-2 [19], reactive oxygen species (ROS) [20,21], co-infecting organisms [22], inflammatory cytokines (e.g., IL-8) and chemokines [23], and activated platelets [24] which could activate neutrophils and induce NETs. The results demonstrated that healthy neutrophils incubated with the plasma of COVID-19 patients had significantly increased NETs.

Production occurred after 2 h (*p* < 0.05) (Figure 1F,G). Moreover, the levels of dsDNA in supernatants (as a marker of NETs production) were significantly elevated in neutrophils treated with the plasma of COVID-19 patients from moderate (*p* = 0.0169) and severe groups (*p* = 0.095) (Figure 1H).

### 2.4. Reduction in NET and mRNA Expression by Enoxaparin Pretreatment In Vitro

**Figure 2:**
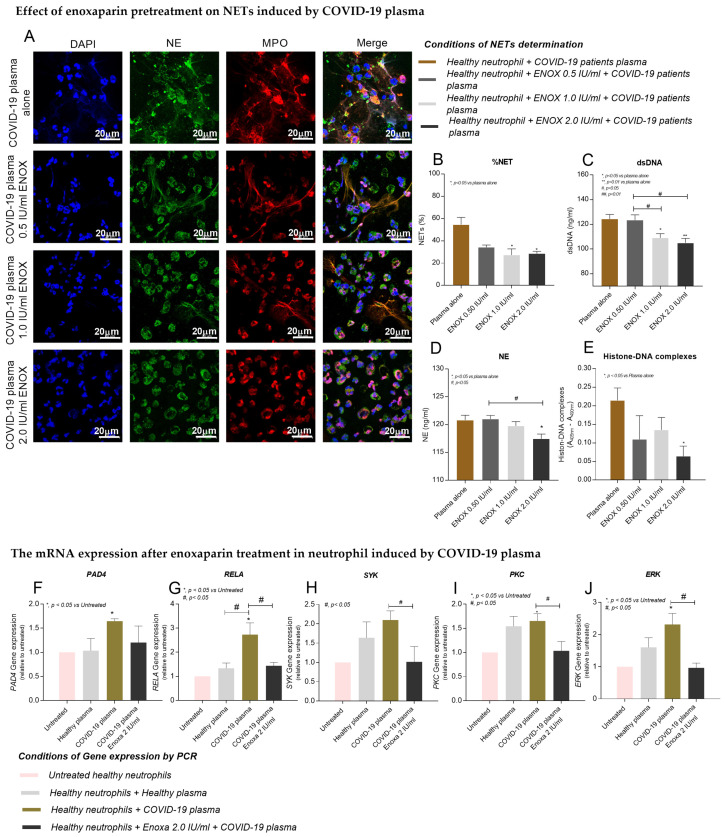
The effect of enoxaparin pretreatment on NETs induced by COVID-19 plasma in vitro. Representative fluorescence images captured by confocal microscope (original magnification 630X with scale bars indicate image size as labelled 20 μm) of isolated neutrophils from healthy controls pretreated with or without enoxaparin (0.5, 1 and 2 IU/mL) and incubated with COVID-19 plasma from severe cases (*n* = 5), stained with DAPI (blue), neutrophil elastase (green) and myeloperoxidase (red), and merged images for NET identification (**A**). The percentages of NETting neutrophils (**B**), the levels of dsDNA (**C**), NE (**D**) and Histone–DNA complexes (**E**) in the supernatants from healthy neutrophils pretreated with or without enoxaparin (0.5, 1 and 2 IU/mL) and incubated with COVID-19 plasma. The mRNA expression levels of *PAD4* (**F**), *RELA* (**G**), *PKC* (**H**), *SYK* (**I**) and *ERK* (**J**) from healthy neutrophils pretreated with enoxaparin (2 IU/mL) and incubated with or without COVID-19 or healthy control plasma (*n* = 5 for all groups) (Supplemental Table S2). Abbreviations: *RELA*, REL-associated protein involved in NF-κB p65 subunit; Protein kinase C, *PKC*; spleen tyrosine kinase, *SYK*; extracellular signal-regulated kinases, *ERK*. (**A**–**E**) (* *p* < 0.05, ** *p* < 0.01 vs. plasma alone and # *p* < 0.05 vs. the indicated group) and (**F**–**J**) (* *p* < 0.05 vs. untreated and # *p* < 0.05 vs. the indicated group).

A previous study demonstrated that neutrophils pretreated with enoxaparin had a decreased formation of NETs [16], and so we investigated whether enoxaparin pretreatment could decrease NETs induced by the plasma of severe COVID-19 patients. The results showed that NETs and the percentages of NET formation induced by COVID-19 plasma were significantly decreased after healthy neutrophils were pretreated with enoxaparin 1 IU/mL (*p* = 0.0395) and 2 IU/mL (*p* = 0.0463) (Figure 2A,B), and the levels of digested dsDNA in collected supernatants were also significantly decreased (*p* < 0.05) (Figure 2C). Moreover, the levels of NE and Histone–DNA complexes were significantly decreased in supernatants after enoxaparin pretreatment (2 IU/mL) (Figure 2D,E, *p* < 0.05).

Previous studies found that the formation of NETs was associated with the expression of inflammatory genes such as *PAD4*, *RELA*, *PCK*, *SYK*, and *ERK* [25,26,27,28,29]. We therefore investigated whether COVID-19 plasma could induce the expression of these genes, and whether their expression was regulated by enoxaparin treatment. The results demonstrated that the levels of *PAD4* (Figure 2F), *RELA* (Figure 2G), *PKC* (Figure 2H), *SYK* (Figure 2I) and *ERK* (Figure 2J) gene expression were signifFicantly increased after healthy neutrophils were incubated with the plasma of COVID-19 patients (*p* < 0.05). Interestingly, enoxaparin pretreatment significantly decreased the expression of these genes in neutrophils incubated with COVID-19 plasma (Figure 2F–J, *p* < 0.05), in parallel with the inhibition of NET formation.

### 2.5. Effects of Enoxaparin Prophylaxis in COVID-19 Patients

**Figure 3:**
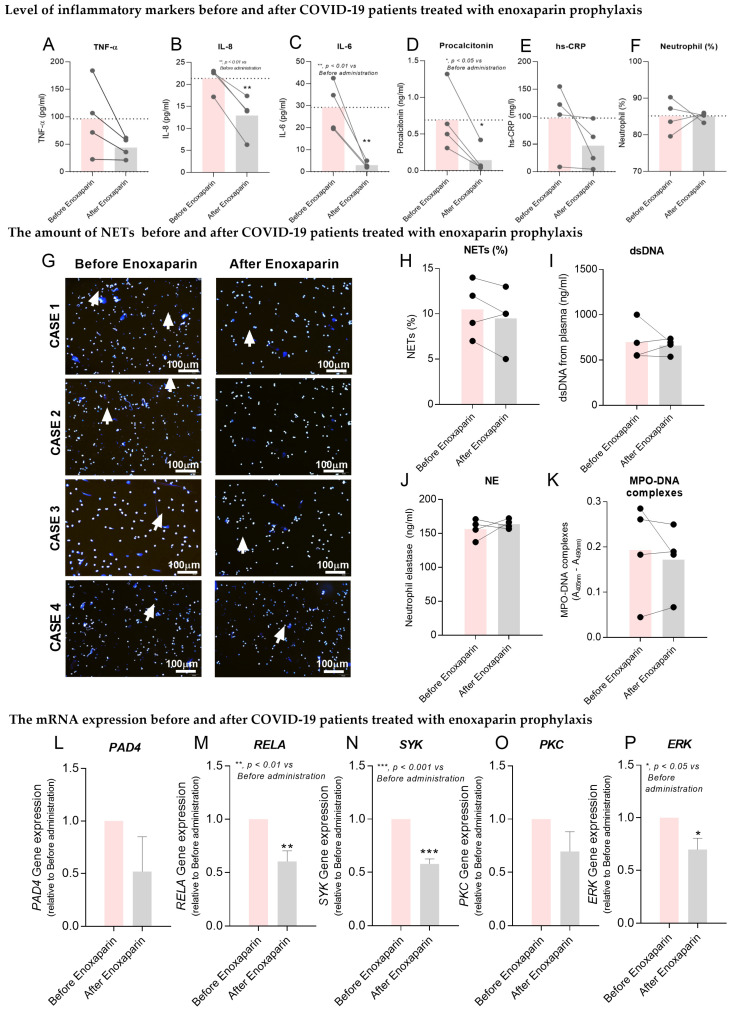
The effect of enoxaparin prophylaxis on NETs and expression of inflammatory markers in COVID-19 patients. Blood was collected from four patients with severe COVID-19 before and 4 h post-prophylactic treatment with enoxaparin, and neutrophil functions and plasma levels of inflammatory mediators were measured. Plasma levels of TNF-α (**A**), IL-8 (**B**), IL-6 (**C**), procalcitonin (PCT) (**D**), and high-sensitivity C-reactive protein (hs-CRP) (**E**) are shown before and after treatment with enoxaparin prophylaxis. (**F**) shows the percentages of neutrophil counts while (**G**) shows representative fluorescence images captured by confocal microscopy (original magnification 200× with scale bars indicate image size as labelled 100 μm) of isolated neutrophils (cases 1–4) stained with DAPI (blue) for NET identification. (**H**) shows the percentages of NETs in isolated neutrophils, while plasma levels of dsDNA (**I**), NE (**J**) and Histone–DNA complexes (**K**) are shown before and after enoxaparin treatment. mRNA expression levels of *PAD4* (**L**), *RELA* (**M**), *PKC* (**N**), *SYK* (**O**) and *ERK* (**P**) in patients’ neutrophils are shown before and after treatment with enoxaparin prophylaxis. *n* = 4 for all measurements (Supplemental Table S3). Abbreviations: *RELA*, REL-associated protein involved in NF-κB p65 subunit; Protein kinase C, *PKC*; spleen tyrosine kinase, *SYK*; extracellular signal-regulated kinases, *ERK*. (* *p* < 0.05, ** *p* < 0.01, *** *p* < 0.001 vs. before enoxaparin administration).

Enoxaparin prophylaxis is recommended in patients with severe COVID-19 infection because these patients have a high risk of developing venous thromboembolism (VTE) [14]. As we found an anti-inflammatory effect of enoxaparin in vitro, we therefore investigated whether prophylactic doses of enoxaparin (40 mg/day) decreased inflammatory markers in the serum of COVID-19 patients. The results demonstrated that the levels of inflammatory cytokines, particularly IL-6 and IL-8, and inflammatory biomarkers for COVID-19 infection (high-sensitivity C-reactive protein and procalcitonin) [30,31], were significantly decreased by 4 h after patients were treated with enoxaparin prophylaxis (Figure 3A–E; *p* < 0.05).

We then measured NET formation in severe cases of patients who received enoxaparin prophylaxis. Although no changes in neutrophil counts (Figure 3F), NET formation (Figure 3G), the percentage of NETs (Figure 3H), and NET products (Figure 3I–K) were observed in patients’ neutrophils after treatment with a prophylactic dose of enoxaparin, the expression levels of inflammatory genes associated with NET formation, particularly *RELA*, *SYK* and *ERK*, were significantly decreased in neutrophils following enoxaparin treatment (Figure 3L–P; *p* < 0.05).

## 3. Discussion

Patients with COVID-19 infection who develop severe pneumonia or ARDS show acute inflammation of the lower respiratory tract, including the lungs. Recent studies have demonstrated that tissues of COVID-19 lungs accumulate inflammatory cells, especially neutrophils, NETs [6,32], and inflammatory cytokines (e.g., IL-8) [17]. Although NETs are an important mechanism for the elimination of pathogens, including SARS-CoV2, the formation of these web-like structures also induces tissue inflammation and vascular thrombosis, leading to serious lung damage in patients with COVID-19 infection [32].

In this study, the COVID-19 patients with severe pneumonia showed systemic inflammation (e.g., elevated liver enzymes and procalcitonin) and coagulopathy, as summarized in Table 2. The plasma levels of NET products (dsDNA, NE, Histone–DNA, and MPO–DNA complexes) were significantly increased, particularly in the severe cases (Figure 1A–D). Moreover, the inflammatory cytokine (IL-8) was also increased in the plasma of these patients (Figure 1E). These findings are consistent with previous studies that showed an increase in NET products and inflammatory cytokines in patients with COVID-19 infection [33,34,35] and suggest that the SARS-CoV2 virus induces systemic inflammation, including NET formation in severe cases. Furthermore, the levels of NET products and IL-8 observed in plasma could be used as biomarkers to determine the disease severity of COVID-19 patients, particularly NE and MPO–DNA complexes, which are mainly produced by neutrophils and more specific to NET formation (Figure 1B,D).

In previous studies, a number of factors were identified in the plasma of COVID-19 patients that could induce NETs and activate neutrophils [10,19,24,36,37]. Therefore, this study investigated whether healthy neutrophils that were incubated with COVID-19 plasma became activated and released NETs in vitro. As predicted, the formation of NETs was increased in neutrophils treated with patients’ plasma, particularly when treated with plasma from moderate or severe cases (Figure 1F–H). This finding confirms that the factors in the plasma of COVID-19 patients contribute to the formation of NETs, and if this occurs in the lower respiratory tract then this can significantly contribute to lung damage.

There are various signaling receptors (e.g., Toll-like receptors, opsonic receptors and cytokine/chemokine receptors) and intracellular networks (e.g., protein kinase pathways, ROS production and MPO, NE and PAD4 enzymes) involved in neutrophil activation that result in the formation of NETs [38]. A recent study has demonstrated that the SARS-CoV2 virus is capable of binding to the angiotensin-converting enzyme 2 (ACE2) receptor on the surface of neutrophils and activates the PAD4 enzyme, causing Histone citrullination with chromatin decondensation, subsequently leading to NET formation. To understand the molecular mechanism responsible for NET production by COVID-19 plasma, we measured the levels of a number of intracellular molecules of neutrophils after incubation with COVID-19 plasma. The results showed that the expressions of genes associated with *SYK* and downstream signals (*ERK-NFκB* and *PKC*), and *PAD4* were upregulated after neutrophils were incubated with patients’ plasma (Figure 2F–J). These findings suggest that neutrophils producing NETs in COVID-19 patients become activated through the PAD4 enzyme and *SYK-ERK-NFκB* and *SYK-PCK* signaling pathways. However, the expression of the ACE2 gene in neutrophils was unchanged after incubation with COVID-19 plasma (data not shown), which is reported to have low levels of SARS-CoV-2 viral load [39].

As NETs are a major contributing factor involved in the pathogenesis of lung injury in COVID-19 patients, the main focus of this study was to search for an adjunct treatment that could be used to prevent NET formation. In a previous study, it was demonstrated that enoxaparin, which has been commonly used as an anticoagulant prophylaxis in COVID-19 patients, blocked SARS-CoV2 entry into the host cells, interfered with the actions of inflammatory cytokines, and prevented neutrophil activation [15,16]. Our study therefore investigated the effects of enoxaparin on neutrophils after incubation with the plasma of COVID-19 patients. Interestingly, it was found that enoxaparin pretreatment at concentrations of 1–2 IU/mL reduced the formation of NETs in neutrophils incubated with plasma from moderate or severe cases in a dose-dependent manner (Figure 2A–E). Moreover, the upregulated genes (*PAD4*, *RELA*, *PKC*, *SYK*, and *ERK*) after incubation with COVID-19 plasma were significantly decreased by enoxaparin pretreatment (Figure 2F–J). Taken together, these data suggest that enoxaparin is potentially a good candidate for the inhibition of NETs in COVID-19 patients with moderate to severe pneumonia, as this drug prevents vascular thrombosis and reduces the formation of NETs. However, it is unknown whether these concentrations (1–2 IU/mL) are over the concentrations for administration to humans, particularly patients with COVID-19 infection. Therefore, the concentration level of enoxaparin in patients with COVID-19 infection after treatment needs to be further investigated. In addition, plasma NE levels could be used as a specific biomarker to determine disease severity. We show that NE levels reflect the level of NETs and are significantly decreased in the supernatants of COVID-19 plasma-treated neutrophils after enoxaparin pretreatment (Figure 2D).

Although enoxaparin significantly decreased NETs in healthy neutrophils incubated with COVID-19 plasma in vitro, the levels of NETs and NET products were not significantly different between patients treated with or without enoxaparin prophylaxis (Figure 3G–K). This result is probably because of the low number of patients recruited (*n* = 4) or, alternatively, that in vivo effects on NET formation had occurred before the 4 h of treatment or were not yet detectable by the 4 h of treatment with this drug. In addition, enoxaparin prophylaxis alone might not be able to completely prevent NET formation in COVID-19 infection as neutrophils isolated from these patients were perhaps already activated by various factors in plasma through different signaling pathways. However, this study confirmed that the anti-inflammatory effect of enoxaparin prophylaxis remained in vivo as the results showed that the levels of inflammatory cytokines (IL-6 and IL-8), inflammatory biomarkers (hs-CRP and procalcitonin) and the expression of genes associated with neutrophil activation (*PAD4*, *RELA*, *PKC*, *SYK*, and *ERK*) were decreased 4 h after patients were treated with enoxaparin prophylaxis (Figure 3L–P). It is possible that, in vivo, the effects of enoxaparin on NET production are secondary to these decreases in inflammatory cytokines. In addition, the effect of enoxaparin on antithrombin III associated with inflammatory genes (e.g., *SYK* or *PAD4*) has not been investigated in COVID-19 patients. Further studies should therefore measure NET production and NET products in the plasma of severe COVID-19 patients after longer treatments with enoxaparin and investigate the association between antithrombin III and these inflammatory genes.

**Figure 4:**
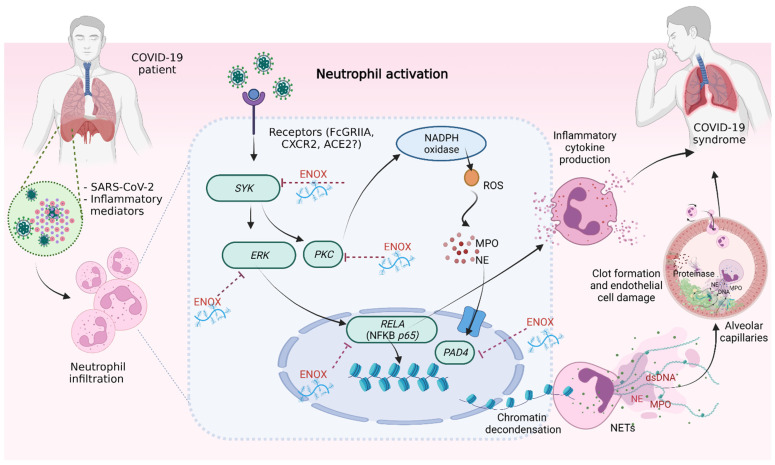
The anti-inflammatory and inhibition of NET production by enoxaparin in COVID-19 infection. SARS-CoV2 virus and inflammatory mediators are recognized by receptors on the cell surface of neutrophils inside the lungs that activate inflammatory cytokine/chemokine release (e.g., IL-8) and NET formation causing lung pathology. Intracellular molecules involved in neutrophil activation include *SYK*, *ERK* and *RELA* (IL-8 production), *PKC* and *PAD4* (NET formation), which are inhibited by enoxaparin prophylaxis. Abbreviations: *RELA*, REL-associated protein involved in NF-κB p65 subunit; nuclear factor kappa B, NF-κB; Protein kinase C, *PKC*; Spleen tyrosine kinase, *SYK*; Extracellular signal-regulated kinases, *ERK*; Protein arginine deiminase 4, *PAD4*; Enoxaparin, ENOX; Angiotensin-converting enzyme 2, ACE2; severe acute respiratory syndrome coronavirus 2, SARS-CoV-2; Reactive oxygen species, ROS; Fc-gamma receptor IIA, FcGRIIA; C-X-C Motif Chemokine Receptor 2, CXCR2; Myeloperoxidase, MPO; Neutrophil elastase, NE; Double-strand DNA, dsDNA.

In conclusion, this study demonstrated that NET products in the plasma of patients could potentially be used as biomarkers to assess the disease severity of COVID-19 infection, particularly NE, which is highly specific to NET formation. Moreover, several factors in the plasma of patients activate neutrophils to produce NETs via *SYK* and downstream signals (*ERK-NFκB* and *PKC*), and *PAD4*. Importantly, this is the first study to show the anti-inflammatory effects of enoxaparin pretreatment, in addition to anticoagulation, and confirmed that this effect persisted in patients treated with enoxaparin prophylaxis. Figure 4 summarizes the anti-inflammatory effects and decreases in NETs by enoxaparin in the pathogenesis of COVID-19 infection. While the n numbers used in this study are relatively low (*n* = 10 for each sub-group of patients), there is consistency in results between our in vitro and ex vivo studies. However, an extended study, particularly analyzing the plasma of patients with severe COVID-19 and correlating these measurements with disease outcomes, would further strengthen our findings. It is also noteworthy that we previously reported spontaneous ex vivo NET formation by neutrophils from sepsis patients [40], and so our findings, particularly the beneficial effects of enoxaparin, may have wider impact on patients with other conditions associated with systemic activation of neutrophils. We propose that enoxaparin prophylaxis is a suitable treatment option in patients with moderate to severe pneumonia because of its dual actions of anticoagulation and anti-inflammation.

## 4. Materials and Methods

### 4.1. Study Design

Thirty patients diagnosed with COVID-19 infection and 10 healthy aged-match controls were randomly recruited from the Thai Red Cross Emerging Infectious Diseases Clinical Center (EID unit), the King Chulalongkorn Memorial Hospital, Thailand. In brief, the inclusion criteria are patients diagnosed with COVID-19 infection and confirmed by FDA-approved positive SARS-CoV-2 polymerase chain reaction (PCR) testing and the quantification of *E* and *ORF1ab* genes of SARS-CoV-2. The exclusion criteria are: (a) patients with other current infections or other respiratory tract infections (e.g., virus, bacteria and fungus) or sepsis, (b) patients with platelet dysfunction or coagulopathy, and (c) patients with other previous infections within 4 weeks before COVID-19 infection. Patients were classified into 3 groups based on the “Living guidance for clinical management of COVID-19” by the World Health Organization (WHO) [41] as: (1) mild (asymptomatic group or symptomatic without evidence of viral pneumonia or hypoxia), (2) moderate (clinical signs of pneumonia but not severe, including SpO2 ≥ 90% in room air), or (3) severe (clinical signs of pneumonia or ARDS) (Supplemental Table S4). The clinical signs and symptoms of pneumonia in COVID-19 patients were confirmed by physical examination and film chest X-ray. To investigate the effect of enoxaparin treatment ex vivo, 4 patients with moderate to severe COVID-19 infection who were treated with enoxaparin prophylaxis (40 mg/day) were randomly recruited and blood samples collected 4 h after the first dose of treatment on day 1 [16,42,43]. This study was approved by Chulalongkorn University Human Research Ethics Committee (IRB No.426/63, 1013/64 and COA No. 738/2020, 87/2022). Written informed consent and/or assent forms were obtained from all donors.

### 4.2. Human Neutrophil Isolation

Whole blood was collected from COVID-19 patients and healthy volunteers by venipuncture into EDTA tubes. Neutrophils (PMN) were isolated as described in our previous studies [40,44,45]. In brief, whole blood was layered onto Polymorphprep™ (Axis-Shield, Oslo, Norway) in a 1:1 ratio and centrifuged for 30 min at 500× *g*-force. The PMN layer, largely comprising neutrophils (>95%), was removed and washed with RPMI 1640 (Thermo Fisher Scientific, Logan, Utah, USA). The cells were resuspended with RPMI 1640 supplemented with 10% heat-inactivated fetal bovine serum (FBS). Contaminating erythrocytes were removed by ammonium chloride lysis buffer. Neutrophil preparations were at least 95% viable and pure, as confirmed by trypan blue exclusion (Sigma-Aldrich, Singapore) and Wright’s Giemsa staining (Biotech, Bangkok, Thailand), respectively.

### 4.3. Detection of NETs

Isolated healthy neutrophils (5 × 10^5^ cells/well) were treated with or without enoxaparin (0.5, 1 and 2 IU/mL, Clexane, Sanofi, Italy) in RPMI 1640 for 30 min at 37 °C [16]. Neutrophils were then placed onto Poly-L- Lysine-coated (Sigma-Aldrich, Singapore) 6 mm glass coverslips, and allowed to attach. The cells were incubated with 10% (*v/v*) plasma of either COVID-19 patients or healthy controls at 37 °C in a 5% CO_2_ incubator. After 2 h of incubation, the coverslips were fixed with 4% (*v/v*) formaldehyde, blocked with Tris Buffered Saline (TBS) in 2% (*w/v*) bovine serum albumin (BSA) (Sigma-Aldrich) and permeabilized by TBS with 0.05% Tween 20 (Sigma-Aldrich, Singapore). The formation of NETs was detected by nuclear morphology, DAPI staining and co-immunofluorescence staining of MPO and NE (Abcam, Cambridge, UK) [44,45]. ProLong antifade medium (Invitrogen) was added, and the cover slips with cells were mounted upside down. Neutrophils isolated from COVID-19 patients treated with enoxaparin were placed onto the glass coverslips directly, incubated and stained for immunofluorescence using the same protocol [40,44,45,46]. NETs were visualized by confocal microscopy.

### 4.4. Quantification of NETs

Neutrophils treated with 10% (*v/v*) plasma of COVID-19 patients or healthy controls, with or without enoxaparin (0.5, 1 and 2 IU/mL, Clexane, Sanofi, Italy) were incubated with 0.1 M CaCl_2_ and 0.5 units (U) of micrococcal nuclease from *Staphylococcus aureus* (Sigma-Aldrich, Singapore) to digest extracellular DNA at 37 °C in a 5% CO_2_ incubator for 10 min. The nuclease reaction was stopped by adding 0.5 M EDTA, and the cell suspension was collected and centrifuged for 5 min. The supernatant was collected and kept at −80 °C [40,44,45,46]. Digested, free dsDNA in the supernatant was measured using Quant-iT™ PicoGreen reagent according to the manufacturer’s instructions (Thermo Fisher Scientific, Paisley, UK). The amount of dsDNA in the supernatant was measured at 480/520 nm on a fluorescent microplate reader (Bio-Tek, Santa Clara, CA, USA) [40,44,45,46].

### 4.5. Quantification of Cytokines and NET Products

Inflammatory cytokines (TNF-α and IL-8) in plasma were measured by enzyme-linked immunosorbent assays (ELISA) (Invitrogen, Waltham, MA, USA). The levels of IL-6, hs-CRP and procalcitonin were quantified by an automated immunoturbidimetric assay (cobas c502, Roche Diagnostics). Neutrophil elastase (NE) level was measured by sandwich ELISA (Abcam270204, Cambridge, United Kingdom). Levels of Histone–DNA complexes were measured by the Cell Death Detection ELISA kit (Roche, Mannheim, Germany).

Levels of MPO–DNA complexes were measured using an adapted protocol from previous studies [47,48]. Briefly, Costar 96 flat-well plates were coated with 1 µg/mL antihuman MPO antibody (Bio-Rad 0400-0002, Oxford, United Kingdom) in coating buffer from the Cell Death ELISA kit, overnight at 4 °C. After washing and blocking, sera or supernatants were added and incubated at room temperature for 90 min. The plate was washed and incubated with HRP-conjugated anti-DNA antibody (from the Cell Death kit), and color was developed with 3,3′,5,5′-Tetramethylbenzidine (TMB) substrate (Invitrogen, Waltham, MA, USA) followed by 2N H_2_SO_4_ to stop the reaction. The absorbance was measured at 450 nm.

### 4.6. Measurement of mRNA Expression

The mRNA levels of the Peptidyl arginine deiminase 4 (PAD4) gene, which is associated with NET formation [25] and neutrophil genes involved in inflammatory signaling—*RELA* (REL-associated protein involved in NF-κB p65 subunit); spleen tyrosine kinase (*SYK*); extracellular signal-regulated kinases (*ERK*) and protein kinase C (*PKC*)—were measured by real-time polymerase chain reaction (RT-PCR) [44,45]. In brief, total RNA was extracted from neutrophil pellets using a FarvoPrep RNA mini kit (Farvogen, Vienna, Australia). The amount of extracted RNA was measured by a NanoDrop OneC Microvolume UV-Vis Spectrophotometer (Thermo Scientific). mRNA levels were quantified by a high-capacity reverse transcription assay (Applied Biosystems, Warrington, UK) and Applied Biosystems QuantStudio 6 Flex Real-Time PCR system with SYBR^®^ Green PCR Master Mix (Applied Biosystems). The relative quantitation was determined using the comparative threshold (2^−ΔΔCt^) as normalized to β-actin (*ACTB*). Primer sequences are shown in Table 5.

### 4.7. Statistical Analyses

Statistical differences among groups were determined by unpaired Student’s *t*-test or one-way analysis of variance (ANOVA) or Kruskal–Wallis test for continuous data and Pearson’s Chi-squared test or Fisher’s exact test for categorical data using STATA software version 15.1 (STATA Corp, College Station, TX, USA). Data are expressed as the mean ± standard error (SE), and differences with a *p*-value of <0.05 adjusted using Benjamini–Hochberg were considered statistically significant.

## Figures and Tables

**Table 1:** Demographic data.

Parameter	Mild (*n* = 10)	Moderate (*n* = 10)	Severe (*n* = 10)	*p-*Value
Gender (Female)	42.6%	33.3%	16.7%	NA
	(NA)	(NA)	(NA)	
Mean Age (yr)	32.71	50.00	48.67	0.29
	(16–49)	(34–71)	(18–84)	
Mean Weight (kg)	62.08	69.00	68.70	0.74
	(49–72)	(60–80)	(85–56)	
Mean BMI (kg/m^2^)	22.39	25.72	26.76	0.34
	(17.78–24.34)	(24.03–27.36)	(23.31–31.22)	
Underlying diseases				
Diabetes	0	2	1	0.263
Hypertension	0	1	1	0.521
Dyslipidemia	0	0	1	0.319
Chronic kidney disease	0	0	2	0.089
Others (e.g., allergy)	0	0	0	0.298

Abbreviations: NA (No data available).

**Table 2:** Complete blood count.

Parameter	Mild (*n* = 10)	Moderate (*n* = 10)	Severe (*n* = 10)	*p-*Value
Red cell count	5.08	4.82	5.05	0.26
	(4.59–5.81)	(4.08–5.35)	(4.11–6.55)	
Hemoglobin	13.41	13.58	13.33	0.78
	(10.2–15.2)	(10.3–15.2)	(12.2–15.5)	
Hematocrit	40.80	40.45	39.48	0.84
	(38.6–45.9)	(32.4–46.9)	(33.6–45.8)	
MCV	80.34	83.97	78.95	0.33
	(66.7–90.8)	(78.2–88.7)	(69.9–84.6)	
MCH	26.41	28.17	26.75	0.88
	(22.2–29.3)	(25.2–31.8)	(23.4–29.7)	
MCHC	32.87	33.60	33.85	0.01
	(32.3–33.3)	(31.8–36.3)	(31.0–36.3)	
RDW	12.94	12.62	12.87	0.96
	(11.6–14.4)	(11.0–14.0)	(12.1–15.2)	
White cell count	4428.57	4795.00	5628.33	0.15
	(2490–7550)	(3410–7650)	(3460–8220)	
Neutrophil%	46.34	60.63	64.48	0.30
	(34.8–58.8)	(45.3–82.1)	(37.8–77.7)	
Neutrophil #	2070.00	2913.33	3786.67	0.26
	(1230–3720)	(1960–5200)	(1310–6390)	
Lymphocyte%	44.14	30.82	26.42	0.17
	(31.8–52.0)	(10.6–44.6)	(9.6–53.2)	
Lymphocyte #	1918.57	1448.33	1353.33	0.82
	(1050–3260)	(360–2110)	(450–1840)	
NLR	1.12	2.86	3.58	0.07
	(0.67–1.85)	(1.02–7.78)	(0.71–8.09)	
Monocyte %	7.67	7.75	8.53	0.40
	(5.4–9.9)	(4.7–10.3)	(5.4–9.4)	
Monocyte #	331.43	385.00	465.00	0.14
	(170–410)	(220–790)	(290–730)	
Eosinophils %	1.46	0.53	0.23	0.10
	(0–2.8)	(0–1.7)	(0–0.8)	
Eosinophils #	65.71	33.33	11.67	0.05
	(0–130)	(0–130)	(0–40)	
Basophils %	0.39	0.27	0.18	0.50
	(0–0.7)	(0–0.7)	(0–0.4)	
Basophils #	14.29	15.00	10.00	0.47
	(0–30)	(0–50)	(0–30)	
Platelet count	205,857.14	207,500.00	199,666.67	0.30
	(34,000–327,000)	(138,000–298,000)	(144,000–282,000)	
MPV	10.68	10.70	11.32	0.52
	(10.3–12.1)	(9.3–11.8)	(10.1–13.3)	

**Table 3:** Blood chemistry.

Parameter	Mild (*n* = 10)	Moderate (*n* = 10)	Severe (*n* = 10)	*p-*Value
AST	18.00	31.83	62.00	0.004
	(12–25)	(18–68)	(26–182)	
ALT	15.86	39.83	45.00	0.04
	(6–38)	(19–81)	(13–109)	
ALP	57.71	66.50	73.33	0.22
	(45–97)	(89–43)	(37–137)	
Total billirubin	0.52	0.57	0.75	1.00
	(0.29–1.05)	(0.28–0.91)	(0.53–1.06)	
Direct billirubin	0.20	0.23	0.36	0.24
	(0.11–0.37)	(0.11–0.39)	(0.18–0.67)	
Serum total protein	8.15	7.80	8.00	0.98
	(7.9–8.4)	(7.6–8.0)	(7.8–8.4)	
Serum albumin	4.30	3.98	3.77	0.40
	(4.1–4.7)	(3.6–4.3)	(3.1–4.4)	
Serum creatinine	0.89	0.78	1.34	0.04
	(0.61–1.39)	(0.50–0.96)	(0.81–2.20)	
Blood urea nitrogen	12.57	10.50	14.33	0.62
	(7–20)	(7–14)	(10–22)	
serum IL-6	NA	NA	1680	NA
	(NA)	(NA)	1680	
serum hs-CRP	NA	43.61	102.85	0.14
	(NA)	(16.95–110.32)	(21.38–230.37)	
serum procalcitonin	NA	0.05	0.28	0.08
	(NA)	(0.02–0.12)	(0.05–1.01)	
serum ferritin	NA	687.65	1464.24	1.00
	(NA)	(346.5–884.1)	(620–4609)	
serum LDH	NA	242.33	375.33	0.23
	(NA)	(200–299)	(198–556)	
d Na	139.33	137.67	136.83	0.38
	(136–142)	(133–142)	(129–141)	
d K	3.55	3.43	3.42	0.88
	(3.2–3.9)	(3.0–3.8)	(2.7–3.8)	
d Cl	106.33	102.50	90.17	0.07
	(104–109)	(99–107)	(21–106)	
d HCO3	25.17	25.33	21.67	0.03
	(23–28)	(23–27)	(12–26)	

Abbreviations: NA (No data available).

**Table 4:** Blood coagulation.

Parameter	Mild (*n* = 10)	Moderate (*n* = 10)	Severe (*n* = 10)	*p-*Value
D-Dimer	NA	364.36	1290.29	0.02
	(NA)	(308.95–411.6)	(794.55–1884.02)	
PT	NA	12.83	13.20	0.01
	(NA)	(11.9–14.3)	(12.8–13.5)	
INR	NA	1.12	1.15	0.01
	(NA)	(1.04–1.25)	(1.12–1.18)	
APTT	NA	25.30	31.40	0.30
	(NA)	(23.0–27.3)	(23.4–56.3)	
Fibrinogen	NA	5.22	4.66	0.41
	(NA)	(4.86–5.91)	(4.02–5.91)	

Abbreviations: NA (No data available).

**Table 5:** Primer sequences.

Target Genes	Forward Primers	Reverse Primers
*PAD4*	5′-CGAAGACCCCCAAGGACT-3′	5′-AGGACAGTTTGCCCCGTG-3′
*NFKB*	5′-GCAGCACTACTTCTTGACCACC-3′	5′-TCTGCTCCTGAGCATTGACGTC-3′
*SYK*	5′-CGTATGAGCCAGAACTTGCACC-3′	5′-CTTTCGGTCCAGGTAAACCTCC-3′
*PKC*	5′-GAGGGACACATCAAGATTGCCG-3′	5′-CACCAATCCACGGACTTCCCAT-3′
*ERK*	5′-TGGCAAGCACTACCTGGATCAG-3′	5′-GCAGAGACTGTAGGTAGTTTCGG-3′
*ACTB*	5′-CACCATTGGCAATGAGCGGTTC-3′	5′-AGGTCTTTGCGGATGTCCACGT-3′

## Data Availability

The data are available from the corresponding author upon reasonable request.

## Supplementary Materials

The following supporting information can be downloaded at: https://www.mdpi.com/article/10.3390/ijms23094805/s1.
