# Supplementary material for: Anti-Inflammatory Effects and Decreased Formation of Neutrophil Extracellular Traps by Enoxaparin in COVID-19 Patients
Source: Int J Mol Sci. 2022 Apr 27;23(9):4805. doi: 10.3390/ijms23094805 (PMC9102387; doi:10.3390/ijms23094805)
Supplement: Supplementary file 1 [file ijms-23-04805-s001.zip › ijms-1685435-supplementary.pdf]

Anti-inflammatory Effects and Decreased Formation of Neutrophil Extracellular Traps by Enoxaparin in COVID-19 Patients

Supplemental Table S1:

Plasma levels of NET products and IL-8 in COVID-19 patients and NET formation induced by COVID-19 plasma.

Figure	Parameter	Healthy control	Mild case	Moderate case	Severe case	*p*-value vs Healthy control
**1A**	**dsDNA**	17.84	23.38	22.73	36.32	< *0.05* at Severe
		(15-21)	(19-26)	(14-31)	(27-39)	
**1B**	**NE**	88.49	105.91	150.50	169.08	< *0.05* at Severe and Moderate
		(59-117)	(57-158)	(140-163)	(161-175)	
**1C**	**Histone-DNA complexes**	0.039	0.033	0.398	1.145	< *0.05* at Severe and Moderate
		(0.037-0.043)	(0.027-0.044)	(0.262-0.612)	(1.049-1.264)	
**1D**	**MPO-DNA complexes**	0.023	0.059	0.128	0.243	< *0.05* at Severe
		(0.020-0.027)	(0.023-0.148)	(0.222-0.119)	(0.183-0.285)	
**1E**	**IL-8**	6.50	8.41	11.14	18.12	< *0.05* at Severe
		(5.20-7.31)	(5.65-14.78)	(6.74-18.26)	(10.00-25.22)	
**1G**	**%NETs counting**	38.58	62.25	73.25	80.75	< *0.05* at Severe and Moderate
		(32-48)	(50-74)	(61-86)	(74-88)	
**1H**	**dsDNA**	90.54	110.66	120.82	121.84	< *0.05* at Severe and Moderate
		(74.06-105.01)	(98.63-135.93)	(106.46-130.05)	(114.64-127.14)	

Supplemental Table S2:

The effect of enoxaparin pretreatment on NETs induced by COVID-19 plasma in vitro.

Figure	Parameter	Plasma alone	ENOX 0.5 IU/ml	ENOX 1.0 IU/ml	ENOX 2.0 IU/ml	*p*-value vs Plasma alone
**2B**	**%NETs counting**	54.1	33.9	26.7	27.5	<*0.05* at ENOX 1.0 and 2.0 IU/ml
		(48.3-61.0)	(32.1-36.2)	(21.8-32.7)	(25.4-30.6)	
**2C**	**dsDNA**	124.37	123.44	109.14	105.06	<*0.05* at ENOX 1.0 and 2.0 IU/ml
		(100.41-150.00)	(97.13-155.92)	(86.60-139.81)	(85.13-126.74)	
**2D**	**NE**	120.86	121.06	119.95	117.56	<*0.05* at ENOX 2.0 IU/ml
		(117.94-126.95)	(117.83-125.54)	(116.87-124.02)	(112.21-120.82)	
**2E**	**Histone-DNA complexes**	0.21	0.11	0.14	0.06	<*0.05* at ENOX 2.0 IU/ml
		(0.11-0.32)	(0.04-0.30)	(0.07-0.19)	(0.05-0.03)	
**Figure**	**Parameter**	**Untreated**	**Healthy plasma**	**COVID-19 plasma**	**COVID-19 plasma+ENOX 2.0 IU/ml**	***p*-value COVID-19 plasma vs COVID-19 plasma+ENOX 2.0 IU/ml**
**2F**	***PAD4* gene expression**	1.00	1.03	1.65	1.20	*Ns*
			(0.60-1.51)	(1.57-1.79)	(0.95-1.23)	
**2G**	***RELA* gene expression**	1.00	1.33	2.73	1.44	*<0.05*

			(1.11-1.78)	(1.55-3.62)	(1.23-1.54)	
**2H**	***SYK* gene expression**	1.00	1.64	2.10	1.02	*<0.05*
			(1.03-2.76)	(1.39-2.50)	(0.65-1.53)	
**2I**	***PKC* gene expression**	1.00	1.54	1.65	1.03	*<0.05*
			(1.88-1.94)	(1.21-1.95)	(0.82-1.23)	
**2J**	***ERK* gene expression**	1.00	1.60	2.31	0.97	*<0.05*
			(1.10-2.23)	(1.62-2.71)	(0.81-1.10)	

Supplemental Table S3:

The effect of enoxaparin prophylaxis in COVID-19 patients on NETs and expression of inflammatory markers in COVID-19 patients.

Figure	Parameter	Before Enoxaparin	After Enoxaparin	*p*-value
**3A**	**TNF-α**	96.17	43.97	*Ns*
		(22.68-183.796)	(21.20-61.52)	
**3B**	**IL-8**	21.36	12.93	*<0.05*
		(17.17-23.04)	(6.30-17.39)	
**3C**	**IL-6**	29.20	3.00	*<0.05*
		(19.5-42.5)	(1.99-4.98)	
**3D**	**Procalcitonin**	0.69	0.15	*<0.05*
		(0.31-1.32)	(0.04-0.42)	
**3E**	**hs-CRP**	97.32	47.24	*Ns*
		(8.56-154.85)	(4.43-96.84)	
**3F**	**Neutrophil (%)**	85.13	85.08	*Ns*
		(79.6-90.3)	(83.3-85.7)	
**3H**	**%NET**	10.5	9.5	*Ns*
		(7.0-14.0)	(5.0-13.0)	
**3I**	**dsDNA**	698.53	659.63	*Ns*
		(553.46-1001.35)	(536.44-735.72)	
**3J**	**NE**	156.76	163.97	*Ns*
		(137.30-170.96)	(156.67-172.17)	
**3K**	**MPO-DNA complexes**	0.19	0.17	*Ns*
		(0.05-0.29)	(0.07-0.19)	
**3L**	***PAD4* gene expression**	1.00	0.52	*Ns*
			(0.19-0.87)	
**3M**	***RELA* gene expression**	1.00	0.604	*<0.05*
			(0.39-0.80)	
**3N**	***SYK* gene expression**	1.00	0.58	*<0.05*
			(0.50-0.67)	
**3O**	***PKC* gene expression**	1.00	0.70	*Ns*
			(0.38-1.02)	

**3P**	***ERK* gene expression**	1.00	0.70	*<0.05*
			(0.50-0.87)	

Supplemental Table S4:

Living guidance for clinical management of COVID-19" by the World Health Organization (WHO)

Group	Definition	Details
Mild	No pneumonia	- asymptomatic group or symptomatic without evidence of viral pneumonia or hypoxia<br>- most experience of mild symptoms are fever, cough, fatigue, anorexia, shortness of breath, myalgias. Other non-specific symptoms, such as sore throat, nasal congestion, headache, diarrhea, nausea, and vomiting
Moderate	Moderate Pneumonia	- clinical signs of pneumonia (fever, cough, dyspnea, fast breathing) but not severe including with $SpO_2 \geq 90\%$ on room air
Severe	Severe pneumonia or ARDS	- clinical signs of severe pneumonia (cough or difficulty in breathing + fast breathing or chest wall indrawing) including $SpO_2 < 90\%$ on room air<br>- chest imaging: (radiograph, CT scan, or lung ultrasound): bilateral opacities, not fully explained by volume overload, lobar or lung collapse, or nodules
